# Operationalizing Access for Chimeric Antigen Receptor T cell Therapies: A Cross-functional Perspective

**DOI:** 10.1016/j.mayocpiqo.2025.100682

**Published:** 2025-12-10

**Authors:** Surya Singh, Carol Greulich, Ariel Perez, Kelly Terrell, Julie Walz-Jensen, Michael D. Dalzell

**Affiliations:** a InformedDNA, St. Petersburg, FL; b CGP Consulting, Cincinnati, OH; c Miami Cancer Institute, Baptist Health South Florida, Miami, FL; d Siteman Cancer Center, Barnes-Jewish Hospital at Washington University School of Medicine, St Louis, MO; e Julie Walz Consulting, Evansville, IN; f ADVI Health, Washington, DC

## Abstract

Chimeric antigen receptor T cell (CAR T) treatment efficacy has been shown to be greater in those who receive timely infusions, while mortality rates increase with each month’s delay in treatment. Yet health care infrastructure constraints, an intricate treatment process, and reimbursement complexities present challenges that affect timely patient access to CAR T therapy. Best practices for decreasing time to treatment are not well established. Autolus Inc convened an expert panel of 3 advisors from established hematopoietic stem-cell transplant centers and 3 advisors with extensive national or regional payer experience to identify operational barriers that contribute to treatment delays as well as potential means for addressing them. Opportunities exist to expand treatment capacity by reducing redundant prerequisites for treatment center certification and through collaboration between established centers and newer centers that need critical expertise to gain accreditation. Aligning clinical criteria are important for improving clinician understanding of the treatment process, facilitating timely referral to treatment centers, and streamlining payer authorization processes. Negotiating financial arrangements is the most time-consuming step of the process before CAR T manufacturing can begin; contracts between treatment centers and payers can help to facilitate timely care, but single-case agreements are necessary for treatment centers and payers without extensive CAR T experience. Single-case agreements should consider each side’s experience and financial exposure. In identifying obstacles to timely care and working through potential solutions, participants developed a genuine appreciation for the interdependence among stakeholders. Recognition of mutual interest is a starting point for cross-functional cooperation.

Cell and gene therapies (CGTs) compose a fast-growing area of cancer treatment and research. As of the end of 2024, more than a dozen of the 43 CGTs approved by the US Food and Drug Administration (FDA)—8 of them chimeric antigen receptor T cell (CAR T) therapies—had hematology or oncology indications.^1^, ^2^, ^3^ Approximately 300 additional CGTs were in late-stage clinical trials for oncologic use.^3^

The rapid pace of approvals has led to predictions about widespread, data-driven adoption of CGT and CAR T therapies. However, capacity constraints and reimbursement complexities present challenges confounding these expectations. In 2024, ∼100 facilities in the United States were certified to offer CAR T therapy.^4^ These centers absorb much of the patient volume, in part because the ever-increasing number of referrals exceeds the growth in certification of new centers of excellence (COE). Moreover, the largest centers have at least some payer contracts in place for CAR T reimbursement, while other centers and payers are obliged to negotiate time-consuming single-case agreements.

Infrastructure and operational bottlenecks increase time to treatment in a patient population whose disease tends to progress rapidly, many of whom have relapsed or refractory disease, and therefore need care as quickly as possible to maximize outcomes. For example, median overall survival in patients with refractory large B-cell lymphoma or who relapsed within 1 year has been estimated at 6.3 months,^5^ and for some acute subtypes of B-cell lymphoma, such as acute lymphoblastic leukemia, overall survival is less than 6 months.^6^ Yet a recent study of access barriers to CAR T therapies found a median time from referral to infusion of 143 days—and during this period, one in every 8 of the 254 patients in this study could not be infused because of disease progression or a decline in clinical status.^7^ Treatment efficacy in patients eligible for CAR T has been shown to be greater in those who receive timely infusions,^8^ whereas mortality rates increase with each month’s delay.^9^

Best practices for decreasing time to treatment are not well established. Thus, there is a need for stakeholders not only to identify operational barriers that contribute to treatment delays but also to develop strategies to address them. To this end, in January 2025, Autolus Inc sponsored a moderated expert panel discussion consisting of 3 advisors from high-volume hematopoietic stem-cell transplant (HSCT) centers and 3 advisors with extensive national or regional payer experience. Members of this panel discussed their own experience with CAR T-related administrative and reimbursement challenges, with the purpose of proposing opportunities for addressing recurring themes. These challenges were previously identified by 2 focus groups Autolus sponsored in the fall of 2024, one involving 15 clinical and administrative decision makers at HSCT centers of excellence and the other involving 15 decision makers from regional and national payers. Participants in the 2024 focus groups were not the same individuals as those who participated in the January 2025 expert panel. To avoid influencing the flow of the conversation or leading the panel to preconceived solutions, Autolus did not participate in the expert panel discussion.

Informed by the findings of the 2024 focus groups, advisors on the January 2025 expert panel discussed real-world implications of the challenges across the payer/provider spectrum, explored avenues for collaboration, and put forth several recommendations for making operational processes more efficient. This paper presents the panel’s observations, insights, and recommendations for collaboration.

It is worth noting that each payer and healthcare system has unique standard operating procedures, philosophies, and cultures, and these differences were evident during this panel discussion. Each organization has its own approach to CAR T-related processes and operations; as such, there was not universal agreement among advisors on this expert panel on every issue, nor was a singular path forward identified. There was, however, a shared desire to enable the best possible patient outcomes and a willingness to work collectively toward efficient operationalization. This paper is intended to encourage stakeholder dialogue, identify areas of common interest, and foster the creation of shared value: what’s beneficial for the patient can also be beneficial for payers and providers.

### CAR T: Operational Challenges and Potential Solutions

Patient access to CAR T therapy involves a complex series of steps that begin several weeks or even months before an infusion.^10^ Not all treatment centers follow the same linear path to infusion, but in general, stages of this process may include:

1. Patient identification through internal or external referral/initial consultation
2. Benefits investigation
3. Prior authorization
4. Negotiation of a financial agreement with the payer
5. Apheresis
6. Manufacturing
7. Lymphodepletion
8. CAR T administration
9. Follow-up care

The aforementioned 2024 focus groups estimated the average time from patient identification to infusion to be 60 to 75 days (range, 35-135 days), based on their own experience, with wide variance in each step of the treatment process (Figure 1). Advisors on the 2025 expert panel noted that these timeframes were similar to their own observations in practice. Working backward from CAR T administration in Figure 1, advisors noted that the time required for administration, lymphodepletion, and manufacturing is largely outside of the control of treatment centers and payers. There was consensus, however, that the steps before manufacturing—from patient identification to apheresis—could be streamlined.

**Figure 1:**
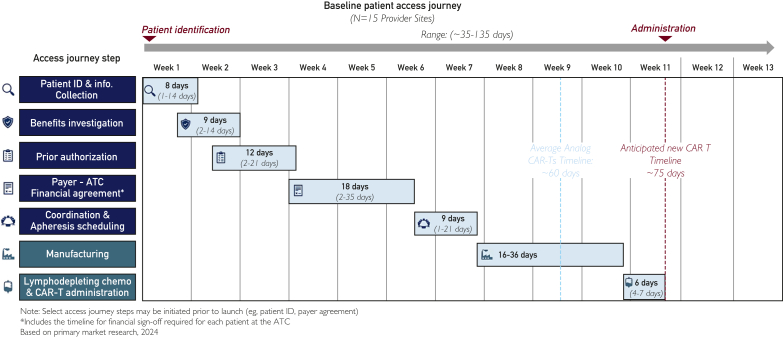
Provider perceptions of time required for stages of CAR T access journey. Informal interviews with clinicians and clinical decision makers at 15 HSCT centers of excellence across the United States determined that the average time from patient identification to infusion was 60 to 75 days. HSCT, hematopoietic stem-cell transplant.

The expert panel discussed four broad issues that contribute to time to treatment challenges: (1) system capacity constraints; (2) clinical and operational complexities; (3) prior authorization processes; and (4) financial agreements. Issues (2) through (4), detailed below, describe advisors’ own experience or opinions of factors that may contribute to variability in the duration of these steps. Issue (1), capacity constraints, is a parallel concern that precedes the first step of the access journey (patient identification and information collection) and overlays the other three issues described here. Capacity constraints cannot be measured in days, but they do influence timely access to care.

With recognition that awareness of the challenges that other stakeholders face is helpful, treatment center and payer advisors explored opportunities to address impediments to timely care inherent in each of these four issues. Ultimately, the group identified several opportunities—again, based on their own experience—for provider–payer collaboration and for making the treatment-access journey more efficient. In the absence of established best practices, these opportunities represent important considerations for moving to action.

#### Capacity Constraints

There is a universally acknowledged need for more treatment centers as demand for CGT and CAR T therapies grows. However, the processes set forth by manufacturers and accreditation agencies that treatment centers must follow for certification are rigorous and time-consuming.

Centers seeking to offer a given therapy must meet an extensive series of manufacturer prerequisites that can take 6 to 8 months to fulfill. Although most prerequisites are similar across therapies, each product and manufacturer have unique requirements. The accelerating pace of FDA approvals adds to the challenges of onboarding and readiness. In 2023, the FDA licensed 6 CGTs; in 2024, 10 CGTs were approved.^2^^,^^3^ For treatment centers willing to participate, the result is an increasing commitment of time, resources, and staff across multiple disciplines.

To ensure sites of care have capacity, proficiency, and CAR T-qualified multidisciplinary teams, a center must also achieve third-party accreditation, such as that from the Foundation for the Accreditation of Cellular Therapy (FACT). Therein lies a conundrum. Payers hesitate to send members to nonaccredited facilities, but accreditation requires clinical expertise built through experience accumulated by administering the therapies via a requisite caseload. As of May 2025, only 155 FACT-accredited sites provided one or more types of immune effector cellular therapy, though the number of centers that offer CAR T therapies may be even lower.^4^^,^^11^ Advisors on the panel unequivocally recognized the need for more accredited centers. As one medical director representing a regional payer put it, “There’s no way the tertiary care institutions can handle the load. We have to figure out how to move at least the cases that are less complicated.”

A silo effect indirectly contributes to capacity constraints. Manufacturers and accreditation bodies have separate processes for validating new centers, and payers have unique policies that influence the flow of patients to them. Advisors were inclined to explore ways to bridge requirements and to introduce operational efficiencies.

There was agreement that redundancy across manufacturers’ prerequisites should be addressed. Some of this could be accomplished by standardizing terminology and by reducing the burden of duplicate documentation. In turn, this may improve efficiency by enabling standardized workflows at treatment centers. Advisors endorsed the work of the American Society for Transplantation and Cellular Therapy 80/20 Task Force, which has proposed 80% of manufacturer requirements be standardized and made 5 recommendations for reducing the burden of documentation:^12^

(1) Eliminate duplication in accreditation and auditing of clinical sites;
(2) Define expectations for the education about and management of CAR T therapy toxicities to potentially replace product-specific risk evaluation and mitigation strategy (REMS) programs;
(3) Streamline current REMS education, testing, and data reporting;
(4) Standardize information technology platforms supporting enrollment, clinical site-manufacturer communication, and logistics of maintaining chain of identity/chain of custody across multiple transportation steps; and
(5) Encourage the use of universal nomenclature by cell therapy manufacturers.

Advisors supported development of a hub-and-spoke training system for helping new centers to gain expertise and experience for accreditation. Under this framework, a large center would enlist fellows and other clinical and administrative staff to provide training and insight to affiliated sites. To develop the volume needed for accreditation, a smaller center could start with patients covered by fee-for-service Medicare, which does not require FACT accreditation. If a large center has standardized processes reporting clear guidance and oversight of smaller centers, it could extend its accreditation to smaller partners, eventually moving some commercial cases to these sites while the COE retains more clinically challenging cases.

To be confident that the treatment center is capable of safely providing this complex service, payers often designate it as a network provider only after it gains FACT accreditation and signs a contract. A network provider may also be considered a COE once it maintains the highest credentialing standards. Treatment center advisors asked whether making standardized outcomes available to payers could be a means for gaining provisional in-network status, even as a center works toward accreditation. Although treatment centers do track some metrics related to CAR T, FACT requirements for reporting cellular therapy infusions to the Center for International Blood and Marrow Transplant Research are not as rigorous as those for transplant programs,^13^^,^^14^ and the metrics themselves must continue to evolve and mature. To this end, payers acknowledged a need for a more comprehensive set of quantitative data across accredited centers. One advisor noted recommendations published by Dandoy et al^15^ in Transplantation and Cellular Therapy in October 2024, which provide a framework for quality review and standardized outcome reporting, may offer acceptable criteria for designation of a COE.

### Future Opportunities: Increasing Capacity

Manufacturers, which play a role in the future of CAR T accessibility, should work collectively to streamline onboarding requirements for treatment centers. Payer and provider communities can share expertise and resources to develop guardrails for certification of new centers.

#### Clinical and Operational Complexities

Currently accredited centers struggle with workflow issues in trying to accommodate the growing demand. Each referral requires a determination of a patient’s CAR T eligibility, which differs among payers. These differences complicate treatment centers’ efforts to collect medical histories and conduct benefits investigations—slowing these processes despite a growing body of knowledge about the urgency of treatment.

As the number of CAR T therapies has expanded and their indications have evolved, some community-based physicians may not yet have relevant experience with these complex therapies and are unfamiliar with clinical eligibility criteria. Some of this may be traced to a mistaken, if well intentioned, belief that patients who are not candidates for HSCT because of age or comorbidities also are not candidates for CAR T. Patient selection for CAR T depends on several factors, such as previous treatments and a patient’s overall fitness for a particular cellular therapy. Determinations of candidacy may be best made by a multidisciplinary specialty team after referral to a center of excellence.^16^

Advisors noted that even among oncologists, knowledge about when to refer patients for evaluation varies. Referring oncologists may underestimate or don’t routinely consider the many administrative steps involved prior to treatment. As a result, too frequently, patients with rapidly progressive terminal conditions are not referred early enough.

Advisors concurred that standardized criteria for clinical acceptability can be helpful on multiple levels. Although each case is clinically unique, standardized criteria may reduce some ambiguity about a patient’s CAR T eligibility, allowing for greater focus on who is at high risk of accelerated deterioration. Payers can use claims data and AI-enabled predictive modeling to identify members who may become candidates for CAR T. Engaging oncologists and treatment centers in this process may enable creation of population-health management models, which in turn can help to clarify which patients are suitable for evaluation. Given that benefits investigations can take up to 2 weeks, identifying potential candidates for CAR T well before a referral occurs carries implications for timely treatment.

### Future Opportunities: Payer–Provider Collaboration

Payers and providers should work collaboratively to define clinical eligibility for CAR T. This effort would enable educational opportunities for referring clinicians and may help to identify eligible patients earlier, ultimately leading to earlier treatment, when the potential for positive outcomes is higher.

#### Prior Authorization Processes

Prior authorization (PA) is a complex and resource-heavy undertaking for both payers and treatment centers. Although acknowledging the burden on treatment centers, payer advisors made it clear that PA is a necessity that serves several purposes: It aligns with evidence-based guidelines to ensure that a service is clinically appropriate; it is an important trigger for verifying benefits; and it initiates notification to downstream entities that a patient will require not only the infusion itself but also any supportive services associated with an episode of care.

At this stage of the access journey, the overarching obstacle is variation in PA requirements from one plan to the next. Information collection becomes a paramount concern as centers determine which prerequisites were fulfilled before a patient was referred so as to avoid time-consuming duplication. As a condition of PA, some payers may request medical information or testing irrelevant to CAR T—requests that are, likely, a carryover from transplant requirements but which, nonetheless, unnecessarily lengthen the process. As put by one clinician advisor, CAR T candidate patients have active disease and are at risk for clinical deterioration, making more than 2 to 3 weeks for PA pretesting unreasonable. Variation also arises when some payers PA to label while others follow the more restrictive inclusion and exclusion criteria of the product’s pivotal clinical trial.

Advisors from 2 treatment centers provided a contrast in how lack of standard PA requirements influences time to treatment through standard operating procedures and workflows. At one center, multiple steps—from determination of pretesting fulfillment to apheresis scheduling—occur simultaneously while PA approval is sought. This advisor estimated the time from patient identification to treatment at ∼70 days. A second center takes a linear path in which each payer’s pretest requirements are determined and executed, and no further steps can begin until PA is granted. Here, time to treatment is closer to 100 days.

There was general agreement among all advisors that most of the clinical criteria required for PA—such as pretests and a member’s treatment history—could be made more consistent across plans in a region. Standardization of clinical criteria could prompt treatment centers to work with their largest commercial payers to create a summary of critical PA approval criteria, which may simplify the collection of patient information and shorten the PA process. When appropriate, referring providers can conduct pretesting locally to help reduce academic centers’ capacity constraints and accelerate the PA process.

Treatment center advisors also described substantial variation in time to PA approval. This was consistent with findings of the 2024 focus groups with clinicians and clinical decision makers, who perceived PA processes require anywhere from 2 to 21 days (Figure 1). Payer decision makers participating in the fall 2024 focus groups provided lower estimates, ranging from 1 to 14 days, depending on whether a second-level PA review was required, and as long as 30 days if an appeal was involved (Figure 2). Advisors on the expert panel were in agreement that the high ends of both ranges were unacceptable.

**Figure 2:**
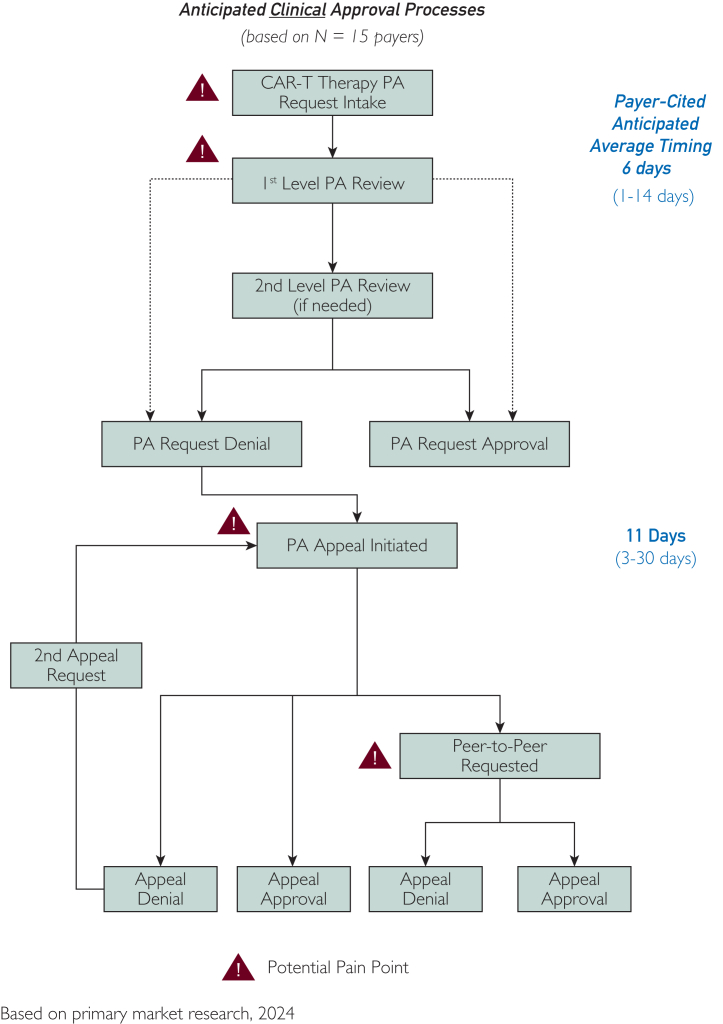
Payer perceptions of time required for prior authorization. In informal interviews, decision makers at 15 national and regional payers estimated that prior authorization required, on average, 1-14 days, and as long as 30 days if an appeal was involved.

Although many payers use the same team to conduct PA for CAR T and HSCT, not all do. When requests for HSCT and CAR T authorization are handled by separate payer teams, gaining approval becomes more complicated. One treatment center advisor noted that this separation was common among several regional payers in the Midwest. As a result, this center is unable to seek simultaneous authorization for either procedure while medical specialists at the center determine a patient’s best course of treatment. If PA is initially requested for transplant but the treatment team later determines that the patient is appropriate for CAR T, the center is required to restart the PA process rather than revise its request—contributing to a delay in time to treatment.

Payer advisors noted that time to a PA determination is often regulated and that delays in reaching a determination may be a function of incomplete fulfillment of requirements. For treatment centers, turnaround time is of utmost importance; the presence of dedicated and experienced case managers may help in this regard and could be considered a best practice. It is possible that, at smaller payers, people without sufficient CAR T experience are reviewing cases; if so, the PA process may need to be more collaborative. One noted his plan has allowed institutions to develop their own clinical criteria for HSCT, subject to plan review and approval. This method has shortened the PA process and simplified payer determinations of whether a patient meets criteria for HSCT set forth by the institution. This may be a viable path for centers with deep experience with CAR T as well.

The inability described by one advisor to seek simultaneous approvals for CAR T and HSCT is not unique. Typically, a payer would request a treatment plan before granting PA for a complex procedure; when a final proposed treatment is yet to be determined, multiple approvals are rarely an option. Some payers do allow for an evaluation authorization in which certain preliminary services for potentially overlapping eligibility criteria are authorized while a center assesses the patient’s candidacy for CAR T or HSCT—a process that may allow the treatment process to begin while a determination is made on a patient’s candidacy for a particular therapy.

### Future Opportunities: Prior Authorization

Standardized clinical criteria may align payer and provider expectations and help to shorten time to a PA. Payers and treatment centers should have dedicated, experienced teams to facilitate information flow and PA turnaround time.

#### Financial Agreements

Development of a financial agreement between a treatment center and a payer is typically the most time-consuming aspect of the access journey prior to the manufacturing stage. Having a CAR T-specific contract in place assures accurate reimbursement and is an important mechanism for timely treatment because it removes the financial agreement as a barrier to access. From an operational standpoint, when a contract is in place, most of what remains to accomplish are the benefits investigation and a PA.

Though advisors universally viewed contracts as preferable, contract development requires a great deal of clinical acumen and practical experience with technological advances in medicine on both sides. Contracts can take as long as a year to negotiate. A new product or indication may require a contract renegotiation. As such, there are instances in which SCAs are necessary. Among payer-related reasons, a payer may be in the process of designating a facility as a COE, or a member’s out-of-network benefit may be inadequate. On the provider side, new programs are difficult to launch from a financial standpoint, and concerns about financial exposure can delay the opening of a program or impede its growth. Some centers—particularly newer centers that are coming to understand their financial exposure—may prefer an SCA, which provides assurance about which services will be covered and what reimbursement can be expected.

As time is of the essence for patients seeking CAR T therapy, payers with CAR T experience can help providers who lack experience with CAR T or familiarity with the negotiation process to reach a general agreement on clinical criteria for CAR T. An interim letter of agreement listing these criteria may make SCA negotiations more efficient.

When SCAs are necessary, terms and definitions should be reused as often as possible to simplify negotiations and reduce their duration. Like all contracts, SCAs have standard elements: member name and identification, diagnosis, procedure to be performed, periods of time required for various clinical interventions, and inclusions and exclusions. The degree to which SCAs can be templated and, in essence, reused varies among treatment centers.

CAR T does not represent the first time this has happened in an area of innovation, and in this case, payers considered analogs for efficiency. One advisor thought it would be feasible for a center to amend HSCT agreements, be they contracts or SCAs, for CAR T. There may be reasons why this is not occurring; however, payers may be hesitant because new-technology outcomes are difficult to measure, or the center may not want CAR T in its COE agreements because it reduces their control over volume and which patients they can accept. These concerns may be alleviated only with time and experience on the part of both stakeholders.

There is wide variance in time to a financial agreement. One advisor recently published an abstract detailing her academic center’s experience with time spent on each step of the access journey. Average time to an SCA was 45 days^17^—considerably longer than the estimates of participants in the fall 2024 focus groups. In those sessions, the 15 payer participants estimated an average time of 7 to 18 days to reach an SCA if the center had negotiated a previous SCA for CAR T, and as long as 29 days for a first SCA for CAR T (Figure 3). By contrast, the 15 COE representatives estimated a range in time to an SCA from 2 days (Medicare) to 35 days (commercial payers) (Figure 1).

**Figure 3:**
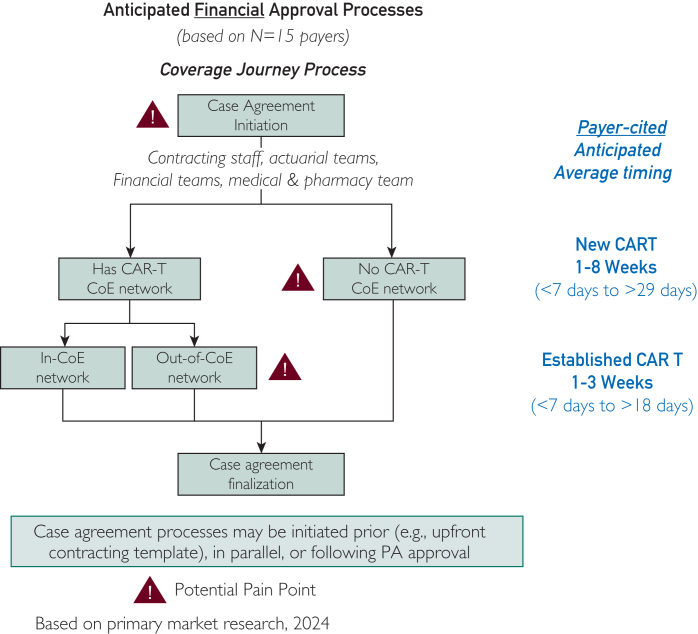
Payer perceptions of time required for SCA development. In informal interviews, decision makers at 15 national and regional payers estimated the average time to negotiate an SCA for CAR T with a COE to be 1 to 3 weeks if the COE had experience negotiating such an agreement. Negotiations for a COE’s first CAR T SCA can take as long as 8 weeks. CAR T, chimeric antigen receptor T cell; COE, center of excellence; SCA, single-case agreement.

Reasons for this variation vary. Experienced providers with established relationships with payers may easily negotiate an SCA within a few days. Newer treatment centers, however, need time and experience to get used to the language of CAR T and the complexities of reimbursement. Centers that draw patients from out of state may have to navigate terms with commercial payers or Medicaid agencies they rarely encounter. Some self-insured employer groups may also have less experience with CAR T or may carve it out altogether.

There was less agreement among payers on the most appropriate form of reimbursement. Because they are inclusive of multiple services, case rates can be simple for commercial payers to administer and, in the opinion of one payer’s representative, prevent the need to micromanage costs. Payers, however, may hesitate to set case rates because of the high-cost nature of CGTs and limited claims data experience for modeling, especially among emerging technologies. These payers may prefer per diems or alternative fee-for-service arrangements, depending, in part, on whether the service is performed on an inpatient or outpatient basis.

Some treatment centers that are still learning where their cost exposure lies may also hesitate to accept a case rate. With a new program, institutional concerns about cost exposure will likely halt the negotiation process, even with dedicated physician champions and patient advocates.

### Future Opportunities: SCA Templates

Although contracts can help to facilitate timely care, SCAs are necessary for treatment centers and payers without extensive CAR T experience. The SCA templates containing standardized information can help to reduce negotiation time. Reimbursement arrangements should consider each side’s experience and financial exposure.

## Discussion

In patients who require CAR T, optimal outcomes rely in part on addressing operational barriers to treatment. There is an evidence-based and well-documented inverse relationship between time to infusion and treatment efficacy/patient outcomes.^8^^,^^9^ Although each payer and health care system has its own approach to CAR T-related activities, there was consensus among members of this expert panel regarding similarities and a shared recognition that these differences do not have to be barriers to timely care.

Time to infusion is a timely topic that will become even more critical to address as the use of CGTs accelerates. The opportunities for stakeholder collaboration described in this paper come at a time when the use of CGTs is expanding but best practices for optimizing time to treatment are not well established. Previously published literature has described barriers to care, though to our knowledge, this paper is the first to bring together decision makers from COEs and payers to discuss real-world experience and to reach consensus on potential paths for overcoming barriers. Nikiforow et al^12^ described, in depth, the work of the ASTCT 80/20 Task Force, which developed recommendations for streamlining documentation requirements as a means for expanding capacity. The report by Nikiforow et al^12^ is necessarily more narrowly focused than this paper, which broaches additional topics and touches on the relevance of the ASTCT recommendations for COEs and payers in the context of their everyday practice. Gajra et al^18^ focused primarily on manufacturing complexities and clinical-care issues. While the authors mentioned PA as an obstacle to care and advocated for a standardized preapproval process, they do not identify specific PA-related issues to be addressed. Feldman^19^ reviewed clinical and administrative barriers to timely treatment, including the apheresis-to-manufacturing stages. As mentioned previously, care delays related to these stages are largely outside of the control of treatment centers and payers. Importantly, a focus on the apheresis-to-manufacturing stages omits the most time-consuming barrier to treatment: financial agreements, which Hu et al^20^ also identified in a study documenting referral to infusion times at two cancer centers. While such empirical evidence is useful for policy decision making, Hu et al do not provide recommendations for improving it. Our paper details the advantages and disadvantages of SCAs and presents potential mechanisms for shortening time to an agreement.

This paper has 2 key limitations. First, the small size of the expert panel—6 individuals—limits the generalizability of the panel’s conclusions. As a matter of practicality, the panel was limited to 6 participants and a moderator to ensure that substantive input was received from all participants. It should be noted that the opportunities for provider–payer collaboration described herein are not presented as best practices; rather, they are directionally relevant paths whose validity should be established through the development of policies and procedures, performance metrics, and outcomes measurement. Second, this panel’s breadth of experience was somewhat narrow, as community oncologists, patient advocates, and other relevant stakeholders were not invited to participate. The concerns of these and other stakeholders—care coordination or disparities based on poverty, race, or distance, for instance—are genuine but are beyond the scope of this paper and have been addressed elsewhere in the literature.^19^^,^^21^^,^^22^ We sought to bring together thought leaders with experience at high-volume treatment centers and payer organizations to discuss real-world barriers to immediate care and avenues for addressing them.

## Conclusion

In working through potential obstacles, participants in this expert panel developed a genuine appreciation for the interdependence among stakeholders. Recognition of this mutual interest is a starting point for cross-functional dialogue. Advisors from payers and treatment centers agreed on the need to collaborate on a regional level to work toward expanding capacity, improve care coordination, refer patients appropriately, and standardize patient selection criteria and financial-negotiation processes to accelerate time to approval.

Manufacturers of CAR T therapies can play a role in helping other stakeholders with their goals for patient and member care. Account teams can help treatment centers understand payers’ access requirements, especially with newer treatment centers seeking to streamline SCA negotiations. Manufacturers can share clinical trial data and approved real-world evidence with payers. They can also provide technical support, tools, and resources that can assist clinical teams and payers with patient support and outcomes management.

All stakeholders—manufacturers, treatment centers, and payers alike—understand the critical importance of time to treatment in terms of patient outcomes. If the systemic barriers and opportunities for overcoming them identified in this paper can be addressed in meaningful ways, improvements in patient outcomes should follow. As the number of approved CGTs continues to grow, ensuring sufficient CGT access continues to grow in criticality. Collaboration among manufacturers, payers, and providers is key to promoting ongoing dialogue, knowledge sharing, and high-quality patient care.

## Potential Competing Interests

All authors received honoraria from ADVI Health for their participation in the expert panel meeting and/or manuscript preparation. In addition, authors of this paper have disclosed the following industry relationships: Dr Perez: Abbvie, Kite Pharma, and Pfizer; Dr Greulich: Anthem, Emerging Therapy Solutions, and Kite Pharma; and Dr Singh: ADVI Health and InformedDNA. The other authors report no competing interests.
